# Multidisciplinary diagnosis and treatment of recurrent follicular dendritic cell sarcoma in abdomen: A case report

**DOI:** 10.1097/MD.0000000000023588

**Published:** 2020-12-18

**Authors:** Chang Qu, Xiaodong Tian, Yongsu Ma, Xuehai Xie, Mingyue Wang, Yujun Dong, Jixin Zhang, Ping Liu, Yinmo Yang

**Affiliations:** a Department of General Surgery; b Department of Dermatology; c Department of Hematology; d Department of Pathology, Peking University First Hospital, Beijing, People's Republic of China.

**Keywords:** Castleman disease, follicular dendritic cell sarcoma, histopathology, paraneoplastic pemphigus, surgery

## Abstract

**Rationale:**

Follicular dendritic cell sarcoma (FDCS) is a rare malignant tumor derived from follicular dendritic cells, and is often associated with Castleman disease. Here we present a rare case of paraneoplastic pemphigus (PNP) with FDCS which required multidisciplinary approach for the diagnosis and treatment.

**Patient concerns:**

A 28-year-old Chinese female had FDCS recurrence, and primary clinical manifestation was PNP.

**Diagnoses:**

PNP with FDCS.

**Interventions:**

The patient received gamma globulin infusion, took anlotinib, and underwent plasma exchange therapy.

**Outcomes:**

The skin lesions recovered and there was no evidence of tumor recurrence.

**Lessons:**

The diagnosis and management of PNP with FDCS require close cooperation among surgeons, dermatologists, hematologists, otolaryngologists, oncologists, radiologists, pathologists, and respiratory doctors. The interesting clinical manifestations of this patient provide a multifaceted approach to the investigation of the interactions among FDCS, Castleman disease, and PNP.

## 1 Introduction

Follicular dendritic cell sarcoma (FDCS) is a rare malignant tumor derived from follicular dendritic cells with morphologic and phenotypic features of FDCs. Most of FDCS can be resected and treated with radiotherapy and chemotherapy.^[1]^ FDCS usually occurs in the lymph nodes, especially in the neck, mediastinum, and axillary region, but also in the extranodal area.^[2]^ Local recurrence can occur in 40% to 50% of cases, and metastasis occurs in about 25% of patients with FDCS. However, retroperitoneal lymph node involvement is extremely rare. About 10% to 20% of FDCS is associated with Castleman disease (CD).^[3]^

Paraneoplastic pemphigus (PNP) is a severe autoimmune blistering disease associated with neoplasms. PNP is most commonly of lymphoproliferative origin and presents a high mortality. It is extremely rare for both FDCS and CD to coexist with PNP. In this case report, we present an interesting case of combined FDCS, CD, and PNP.

## 2 Case report

A 28-year-old Chinese female presented with 2-year history of tumor resection on the greater omentum, and histopathologic examination showed typical features of FDCS. Histopathology of the abdominal mass showed a large number of abnormal cells in the lymph nodes, most of them were spindle cells and polygonal cells. The tumors were arranged in bundles, mixed with scattered small lymphocytes, which were malignant tumors. Immunohistochemistry showed that tumor cells of the sarcoma were LCA++, CD21+++, NSE+, S-100-/plasma+, CD56-, CD117-, DOG1-, CD34-, CD99-, CD1a-, PLAP-, and Inhibin-.

**Figure 1:**
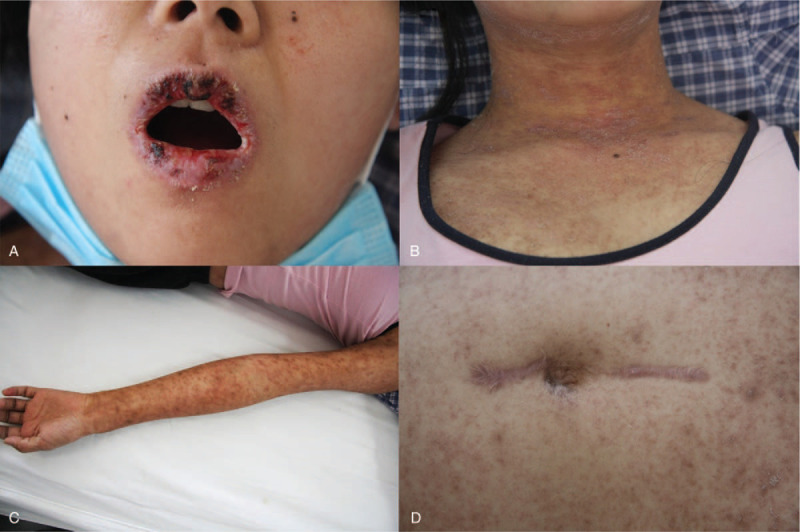
Skin lesions of the patient. (A and B) Vesiculobullous mucocutaneous eruptions around the lips and neck. (C) Crusts surrounded by erythema on the extremities. (D) Abdominal skin was scattered in the rash with scales, and an 8 cm scar was visible in the middle of the abdomen. Written informed consent was obtained from the individual for the publication of this image.

Interestingly, she did not have any discomfort in addition to the abdominal mass at that time. After surgical removal of the mass on the greater omentum, no follow-up treatment was performed. Unfortunately, she began to suffer from painful erosions on the buccal mucosa as well as polymorphic cutaneous lesions including erythemas and erosions. She later developed conjunctivitis associated with blurred vision and atypical targetoid lesions with crusts surrounded by erythema scattered on the trunk and extremities even vulva and perianal region (Fig. 1).

**Figure 2:**
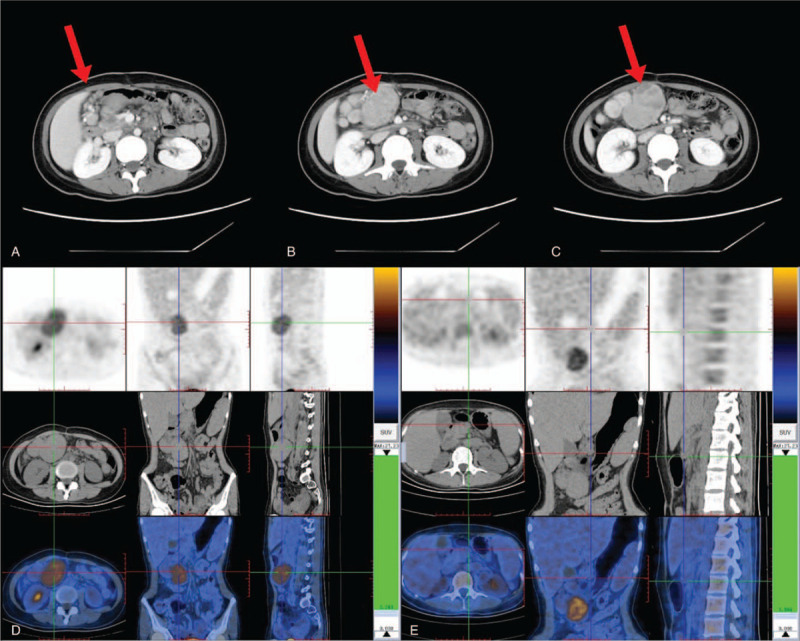
Radiological findings of the tumor. (A) Above the head of the right upper abdominal pancreas, multiple soft tissue nodules and masses of different sizes were found around the right gastroepiploic vein. (B) The margin of the mass was clear, and the longest diameter was about 6.4 cm. (C) Enhanced scan showed marked heterogeneous enhancement of the mass. (D) The right side of abdominal cavity had multiple soft tissue density nodules and tumors, and the glucose metabolism increased. (E) Peripancreatic lymph node enlargement and slight increase of glucose metabolism. Written informed consent was obtained from the individual for the publication of this image.

However, during 6 months there was no clear diagnosis in the local hospital until she reached the intraperitoneal mass because of extreme weight loss (about 20 kg). For further diagnosis and treatment, the patient was treated in our hospital. Direct immunofluorescence of biopsy specimen from skin lesion showed deposition of Immunoglobulin G (+) and C3 in the intercellular region of epidermal cells mainly in the suprabasal region. Linear deposition of IgG was also observed in dermoepidermal junction suggestive of PNP. Laboratory examinations revealed potential bacterial infection (procalcitonin 2.16 ng/mL; normal <0.05 ng/mL) and high-sensitivity C-reactive protein was thirty-five times higher than normal. Elevated tumor markers suggested potential tumors in various organs (alpha-fetoprotein 29.77 ng/mL; cancer antigen (CA)125 37.72 U/mL; CA24–2 23.32 U/mL). Serum immunoassays showed Desmoglein 3 antibody 82 U/mL (normal <20 U/mL) and interleukin-6 315.75 pg/mL (normal <6.4 pg/mL). The determination of serum complement and subclass of IgG was negative. A diagnosis of PNP was made. We found a 6 cm diameter tough mass in the right upper abdomen, and the boundary was clear. Moreover, computed tomography (CT) detected multiple soft tissue density nodules and masses around the right vein of the gastric retina above the right upper abdomen of the pancreatic head. The boundary was clear, and the largest one was about 6.4 cm long. The enhanced scan showed obvious uneven enhancement (Fig. 2).

To further assess tumor growth, we recommended positron emission tomography (PET)-CT. An 2-deoxy-2-[fluorine-18]fluoro-D-glucose (^18^F-FDG) PET/CT was performed to detect the underlying tumors associated with PNP. We observed high ^18^F-FDG uptake in right upper quadrant mesenteric and other lesion retroperitoneal around the pancreas on maximum intensity image. On axial and coronal view of CT, many heterogeneous soft tissue masses were found below the duodenum horizontal section, measuring 61 mm × 42 mm × 63 mm, and PET and PET/CT fusion images demonstrated homogeneous ^18^F-FDG avidity (Maximum Standard Unit Value [SUVmax] 4.2). The liver was significantly enlarged, the contour was smooth, the proportion of the liver was normal, and the density and radioactivity distribution in the liver parenchyma were normal (SUVmax 2). There was no significant increase in FDG uptake in lymph nodes next to the abdominal aorta with multiple short diameters less than 10 mm. The spleen was obviously enlarged, and radioactive distribution was slightly high (SUVmax 2.3). The pancreas was normal. We detected multiple small lymph nodes in the Ib area of the left neck, II area of bilateral neck, bilateral axillary fossa and bilateral groin area, and some areas had slightly increased FDG uptake (SUVmax 1. 2–2.2).

**Figure 3:**
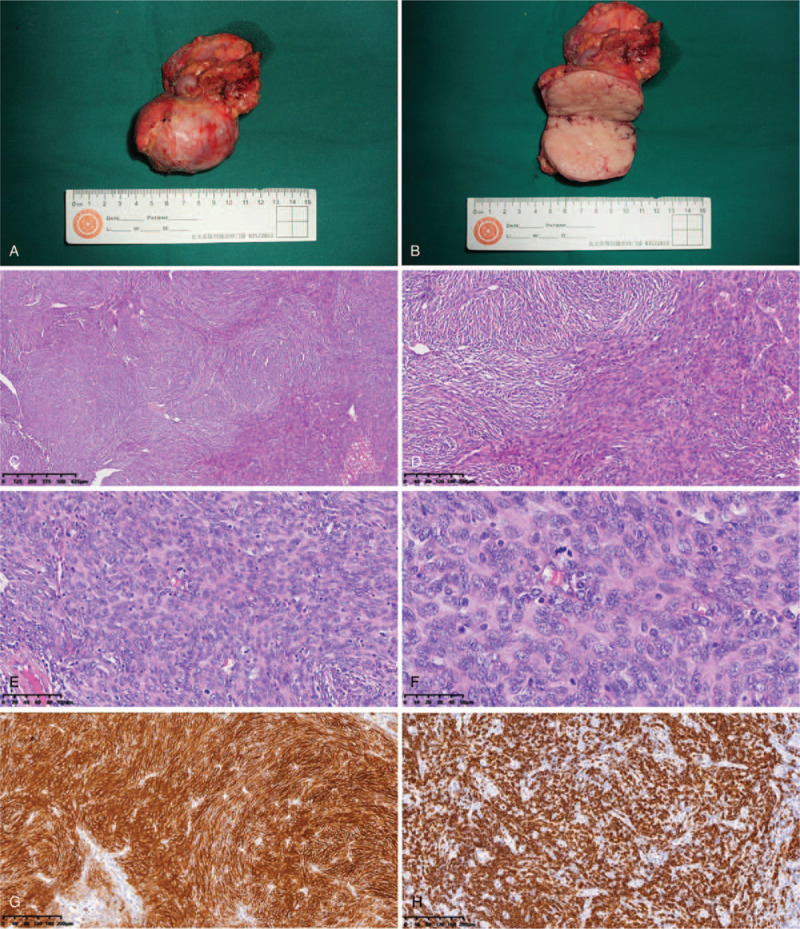
Pathological findings of the tumor. (A and B) Overall and cross-sectional view of the mass. (C) Multinodular fused mass (H&E ×40). (D) Dense proliferation of focal spindle cells in lymphoid tissue (H&E ×100). (E) Some of the tumors were surrounded by a small amount of residual lymph node components, with atrophy of lymphoid follicles, peripheral lymphocyte surroundings, and invasion of small vessels (H&E ×200). (F) The nuclei of spindle cells showed long rods, partly elliptic, with small nucleoli (H&E ×400). (G) Tumor cells diffusely expressed CD21 (Immunohistochemistry ×100). (H) Tumor cells diffusely expressed CD20 (Immunohistochemistry ×100). Written informed consent was obtained from the individual for the publication of this image. H&E = hematoxylin and eosin, IHC = Immunohistochemistry.

For treatment, first she took steroids systemically and took externally applied medicines for the skin. For abdominal masses, we did exploratory laparotomy under general anesthesia. To prevent acute complications, gamma globulin neutralizing antibodies were used during the perioperative period. The mass was located between the 2nd and 3rd layers of the greater omentum. The upper edge of the pancreas was separated from the upper edge of the tumor, and the tumor was isolated from the transverse mesenteric membrane and completely removed. Then we removed the mass around the hepatic artery and dissected it (Fig. 3A and B).

Pathological findings showed that abdominal mass was FDCS with CD-like appearance. Immunohistochemistry results were: lymphocyte CD20+, CD3+; follicular dendritic cells CD21++, CD23++, CD31-/vascular++, HHV8-; plasma cells CD138+, Kappa+, Lambda+. FDCS was found in the superior pancreatic lymph node, and immunohistochemistry results were: follicular dendritic cells CD21++, CD23++, CD35 focal+, S-100-, Ki67 40% (Fig. 3C–H). No tumors were found in the omentum. Genetic testing of the resected tumor showed no mutations of clinical significance.

**Figure 4:**
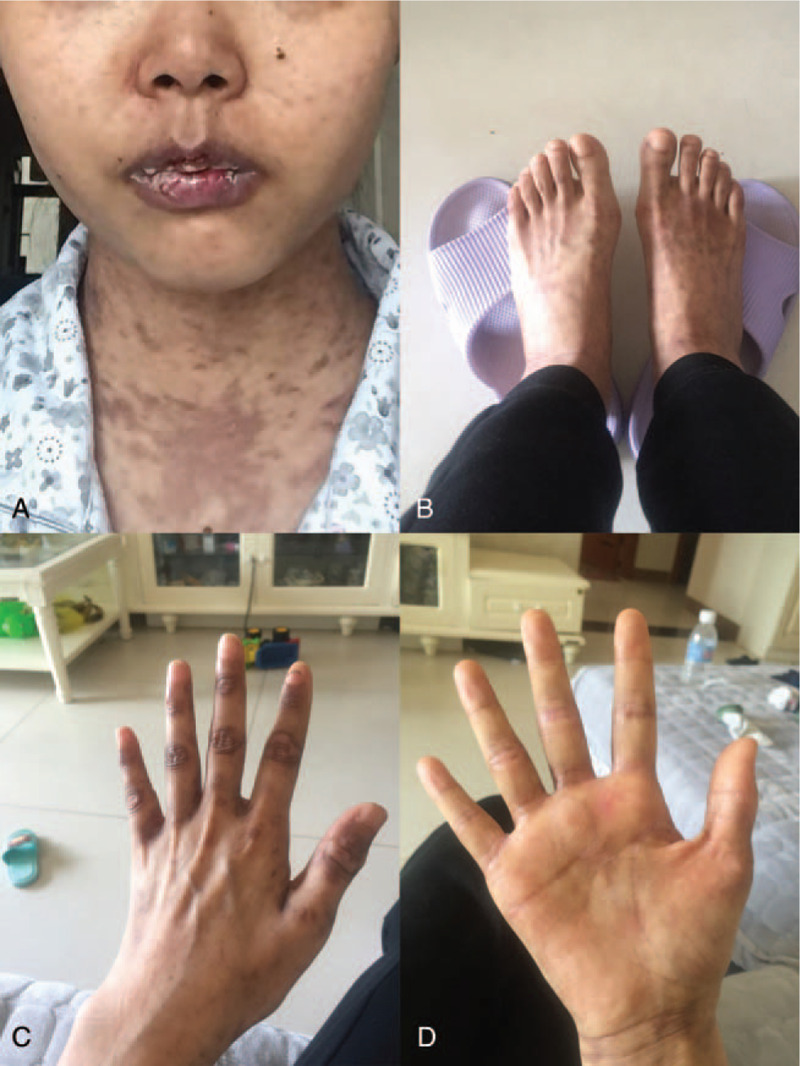
Improvement of skin lesions. (A) Residual pigmentation of skin around the body, oral lesions have improved but not fully recovered. (B, C, and D) Skin lesions of both feet and hands were healed and melanin deposits remained. Written informed consent was obtained from the individual for the publication of this image.

After operation, the patient was discharged successfully, but her skin damage did not improve significantly after surgery. The erythema and erosion of the trunk and limbs were still the same as those before surgery. After discharge, oral methylprednisolone (48 mg/d), infusion of gamma globulin, along with intermittent plasma exchange treatment were used. A small molecular multi-target tyrosine kinase inhibitor Anlotinib was added. The skin lesions were gradually controlled, and the original lesion was gradually healed (Fig. 4). Then the dose of methylprednisolone was gradually reduced to 32 mg/d. CT scan 4 months after the surgery showed that the right axillary lymph nodes were enlarged, but no enlarged lymph nodes were seen in the abdominal cavity. At 6 months after surgery, the skin damage was significantly improved, and the dose of methylprednisolone was reduced to 24 mg/d. Because the symptoms of pemphigus in patients tended to worsen gradually, we recommended plasma exchange therapy to control the symptoms of PNP. At the same time of plasma exchange and gamma globulin infusion, patient took anti-angiogenesis drug Anlotinib. At the third month of follow-up, the skin damage basically healed, presenting as pigmentation, but no significant improvement was observed in oral mucosa.

The patient was re-examined by CT in the local hospital, showing clusters of axillary lymph nodes with a larger diameter of about 0.8 cm and moderate enhancement. At 5 months after the operation, the patient was reviewed again in the local hospital. The results still showed original axillary lymph node enlargement. However, the new lymph nodes behind the jugular vein sheath were enlarged. At 8 months after surgery, the patient's skin recovered well and healed. There were still destroyed mucous membranes in the mouth but significantly relieved. Due to long-term oral cortisol, the patient's face looked like full moon face, and we gradually reduced hormone dose as appropriate. Because the lymph nodes were small and no puncture biopsy has been performed, we still follow-up the patient.

## 3 Discussion

PNP is an autoimmune blistering and erosive mucocutaneous disease associated with neoplasia, and is most commonly of lymphoreticular origin. An alternative term paraneoplastic autoimmune multiorgan syndrome has been proposed to define a condition for severe and often fatal pulmonary involvement and the deposition of autoantibodies in different organs, and for the lesions that resemble pemphigoid, erythema multiforme, graft-versus-host-disease, and lichen planus as well as classic pemphigus.^[4]^

PNP has cancer-related characteristics, including gastric cancer, lung cancer, and colon cancer. In addition, we reported common B-cell lymphoma and hematological malignancies with PNP.^[5]^ Up to 84% of the cases of PNP are caused by hematological tumors or diseases. Lymphoproliferative diseases are the most common diseases associated with PNP. Non-Hodgkin lymphoma is the most common associated tumor (38.6%), followed by chronic lymphoblastic leukemia (18.4%), CD (18.4%), thymoma (5.5%), Waldenstrom macroglobulinemia (1.2%), Hodgkin lymphoma (0.6%), and monoclonal gamma globulin disease (0.6%). In addition, epithelial cell carcinoma (8.6%) and mesenchymal cell sarcoma (6.2%) are associated with PNP.^[6]^

FDCS is a rare tumor derived from follicular dendritic cells. Follicular dendritic cells are non-lymphatic, non-phagocytic helper cells of the lymphatic system, and play an indispensable role in development. In the specimens of the patients after operation, we observed the residual Castleman-like morphology of the tumors, which provided clue to the possible development of the tumors from CD. As for the mechanism of CD induced PNP, it has been suggested that autoantibodies secreted by Castleman tumors play a key role.^[7–9]^ This hypothesis seems to be supported in this case, where CD may trigger both FDCS and PNP.

This case is special because the patient did not have PNP when she first suffered from FDCS. Two years later, the patient suffered from PNP with abdominal tumors. The pathological findings of the tumors were FDCS with Castleman-like manifestations. Because the spindle cells of FDCs occupy almost all of the tumors, only a small number of Castleman-like manifestations remain. It is reported that some FDCS appear in CD lesions and some are accompanied by the lesions.^[10]^ In fact, it is suggested that FDCS comes from the deterioration of CD, so FDCS may have some radiological characteristics of CD. In our case, we observed a large lobulated soft tissue mass with blood vessels. Perhaps the tumors in the abdominal cavity of the patient evolve from CD and gradually transform into FDCS, and PNP may be caused by CD.

The recovery of skin lesions and oral mucosa is not ideal after surgical removal of tumors. Because FDCS behaves more like low-grade sarcoma than lymphoma, complete surgical resection is considered the preferred treatment; however, due to the rarity of the disease, the role of adjuvant therapy (chemotherapy or radiotherapy) remains unclear. Corticosteroids are still considered as the first-line treatment for PNP. However, steroids can only improve skin lesions. In fact, one of the most important clinical features of PNP is the poor response of mucosal lesions to most treatments. Nevertheless, large doses of prednisolone are still recommended as first-line treatment. Prednisolone and other drugs, including azathioprine, cyclosporine, mycophenolate mofetil, cyclophosphamide, intravenous immunoglobulin, and plasma, have been reported to be effective in combination.^[11]^ Rituximab has also been reported to be effective against these diseases.^[12,13]^

Because of different clinical characteristics, PNP with FDCS is still a challenge for the clinicians. In addition, the diagnosis and management of PNP with FDCS require the cooperation among surgeons, dermatologists, hematologists, otolaryngologists, oncologists, radiologists, pathologists, and respiratory doctors. The interesting clinical manifestations of this patient provide a multifaceted approach to the investigation of the relationships among FDCS, CD, and PNP.

## Author contributions

CQ, XT, YM, XX, MW, YD, JZ and PL analyzed the case and YY designed the study. All authors participated in writing and approved the manuscript.

## References

[1] Chen T Gopal P Follicular dendritic cell sarcoma. Arch Pathol Lab Med 2017;141:596–9. 28353378 10.5858/arpa.2016-0126-RS

[2] Baghmar S Kumar S Gupta SD Follicular dendritic cell sarcoma with paraneoplatic pemphigus: rare case and a brief review of literature. Indian J Med Paediatr Oncol 2013;34:317–9. 24604965 10.4103/0971-5851.125255 PMC3932603

[3] Chan JK Fletcher CD Nayler SJ Follicular dendritic cell sarcoma. Clinicopathologic analysis of 17 cases suggesting a malignant potential higher than currently recognized. Cancer 1997;79:294–313. 9010103

[4] Anhalt GJ Kim SC Stanley JR Paraneoplastic pemphigus. An autoimmune mucocutaneous disease associated with neoplasia. N Engl J Med 1990;323:1729–35. 2247105 10.1056/NEJM199012203232503

[5] Wieczorek M Czernik A Paraneoplastic pemphigus: a short review. Clin Cosmet Investig Dermatol 2016;9:291–5. 10.2147/CCID.S100802 PMC5042195 27729809

[6] Paolino G Didona D3 Magliulo G Paraneoplastic Pemphigus: insight into the autoimmune pathogenesis, clinical features and therapy. Int J Mol Sci 2017;18:E2532. 29186863 10.3390/ijms18122532 PMC5751135

[7] Bowen WB Lewis JJ Filippa DA The management of unicentric and multicentric Castleman's disease: a report of 16 cases and a review of the literature. Cancer 1999;85:706–17. 10091744 10.1002/(sici)1097-0142(19990201)85:3<706::aid-cncr21>3.0.co;2-7

[8] Kartan S Shi VY Clark AK Paraneoplastic pemphigus and autoimmune blistering diseases associated with neoplasm: characteristics, diagnosis, associated neoplasms, proposed pathogenesis, treatment. Am J Clin Dermatol 2017;18:105–26. 27878477 10.1007/s40257-016-0235-z

[9] Wang J Zhu X Li R Paraneoplastic pemphigus associated with Castleman tumor: a commonly reported subtype of paraneoplastic pemphigus in China. Arch Dermatol 2005;141:1285–93. 16230567 10.1001/archderm.141.10.1285

[10] Hwang SO Lee TH Bae SH Transformation of Castleman's disease into follicular dendritic cell sarcoma, presenting as an asymptomatic intra-abdominal mass. Korean J Gastroenterol 2013;62:131–4. 23981949 10.4166/kjg.2013.62.2.131

[11] Hertzberg MS Schifter M Sullivan J Paraneoplastic pemphigus in two patients with B-cell non-Hodgkin's lymphoma: significant responses to cyclophosphamide and prednisolone. Am J Hematol 2000;63:105–6. 10.1002/(sici)1096-8652(200002)63:2<105::aid-ajh14>3.0.co;2-c 10629583

[12] Kwatra SG Boozalis E Pasieka H Decreased recognition of paraneoplastic pemphigus in patients previously treated with anti-CD 20 monoclonal antibodies. Br J Dermatol 2019;180:1238–9. 30648258 10.1111/bjd.17577

[13] Imataki O Tamai Y Abe Y A case of follicular lymphoma complicated with lethal pemphigus. Gan To Kagaku Ryoho 2006;33:1677–80. 17108741

